# The Potential of Stem Cells in the Treatment of Skeletal Muscle Injury and Disease

**DOI:** 10.1155/2012/282348

**Published:** 2011-12-19

**Authors:** S. MacLean, W. S. Khan, A. A. Malik, S. Anand, M. Snow

**Affiliations:** ^1^University of Manchester, Manchester M13 9PL, UK; ^2^Institute of Orthpaedics and Musculoskeletal Science, Royal National Orthopaedic Hospital, Stanmore, Middlesex HA7 4LP, UK; ^3^Spinal Deformity Unit, Royal National Orthopaedic Hospital, Brockley Hill, Stanmore, Middlesex HA7 4LP, UK; ^4^Stepping Hill Hospital, Stockport SK2 7JE, UK; ^5^Department of Sports Inury, Royal National Orthopaedic Hospital, Birimingham B31 2AP, UK

## Abstract

Tissue engineering is a pioneering field with huge advances in recent times. These advances are not only in the understanding of how cells can be manipulated but also in potential clinical applications. Thus, tissue engineering, when applied to skeletal muscle cells, is an area of huge prospective benefit to patients with muscle disease/damage. This could include damage to muscle from trauma and include genetic abnormalities, for example, muscular dystrophies. Much of this research thus far has been focused on satellite cells, however, mesenchymal stem cells have more recently come to the fore. In particular, results of trials and further research into their use in heart failure, stress incontinence, and muscular dystrophies are eagerly awaited. Although no doubt, stem cells will have much to offer in the future, the results of further research still limit their use.

## 1. Introduction

Skeletal muscle is the most abundant tissue of the human body, it is highly dynamic and has the ability to regenerate. Loss of skeletal muscle through trauma, tumour ablation, or prolonged denervation is a common clinical challenge.

Despite the ability of muscle fibres to regenerate, muscle function is often compromised after injury due to the formation of dense fibrotic scar tissue. This may be induced by a rise in TGF-B1 and IGF-1, causing postnatal muscle-derived stem cells (MDSCs) and other myogenic cells to differentiate into myofibroblasts, producing type 1 collagen, the major component of fibrotic tissue [1–5]. Although some studies have looked at the effects of blocking components of the inflammatory cascade, such as agents that block TGF-B1, these treatment methods have potential deleterious side-effects [6–8]. 

Skeletal myopathies present a different challenge. Muscular dystrophy is caused by a defective gene encoding dystrophin, which links the extracellular matrix in muscle to the intracellular cytoskeleton. As skeletal muscle is composed of large multinucleated fibres whose nuclei cannot divide, cell therapy has to restore gene expression in hundreds of millions of postmitotic nuclei [9]. 

Clinical application of skeletal muscle engineering in human subjects thus far has been limited, with clinical trials on humans concentrating on cardiac disease, stress incontinence of the bladder, and muscular dystrophies. This in part is due to the challenges of transferring ex vivo to in vivo tissue engineering. It is also due to the complexity of the microenvironment needed to ensure stem cell integration and function. This review will focus on the potential of stem cells for skeletal muscle engineering; their sources, microenvironment, and clinical applicability.

## 2. Anatomy

The formation of skeletal muscle begins during the fourth week of embryonic development as specialised mesodermal cells, termed myoblasts, begin rapid mitotic division. The cytoplasmic fusion of myoblasts forms what is known as myotubes, and by week nine of development these can be identified as multinucleate skeletal muscle cells, termed muscle fibres. By month five, the muscle fibres are accumulating protein filaments important in muscle contraction. As growth of the muscle fibres continues, aggregation into bundles occurs, and by birth myoblast activity has ceased.

The electromicroscopic structure of a muscle fibre reveals a structured longitudinal arrangement of proteins—named myofilaments. These are arranged in groups within the muscle fibre known as myofibrils. The major myofilaments are actin, and myosin. These form functional sub-units known as sarcomeres. Muscle contraction on a subcellular level is a complex process in the sarcomere involving influx of calcium ions into the muscle fibre, and interaction between myosin, actin and the proteins troponin and tropomyosin. This results in the myofilaments sliding relative to one another, generation of ATP, shortening of the sarcomere, and subsequent contraction of the muscle belly.

Contracting muscle fibres would be ineffective if they worked as isolated units. Each fibre is bound to adjacent fibres to form bundles. An accumulation of muscle bundles forms the muscle belly itself. Supporting connective tissue is present, surrounding, and within the muscle. The endomysium surrounds individual fibres, the perimysium encloses the fascicles, and the epimysium surrounds the muscle belly itself.

## 3. Sources

Stem cells may be totipotent, pluripotent, or multipotent, depending on tissue type. Totipotent cells form all the cells and tissues that contribute to the formation of an organism. Only the embryo itself is totipotent. Pluripotent cells can form most cells of an organism from all three germ cell layers. Embryonic stem cells present in the fertilised oocyte, zygote, and morula [10]. Pluripotent cells have the ability to expand in vitro almost indefinitely and form tissues from ectoderm, mesoderm, or endoderm. Concerns about tumour formation in vivo and ethical concerns regarding their harvest have thus far restricted their use.

Multipotent cells form a number of cells or tissues that are usually restricted to a particular germ layer. Multipotent cells are derived from specific tissue compartments in the adult. The two main types of multipotent stem cell are haemopoietic and mesenchymal type, both usually derived from adult bone marrow, but occasionally from fat, skin, periosteum, and muscle, as described below. Mesenchymal stem cells (MSCs) are multipotent, capable of differentiating into several connective tissue types including osteocytes, chondrocytes, adipocytes, tenocytes, and myoblasts [11]. Mesenchymal stem cells have the advantage of being easily obtainable in adult tissue, and with the appropriate microenvironment, can differentiate into various target tissue types.

For skeletal muscle engineering, most research thus far has focused on the satellite cell. The satellite cell was first described by Mauro in 1961, who observed them as mononuclear cells between the basal lamina and plasma membrane (sarcolemma) of the muscle fibre [12]. In response to injury, satellite cells are activated, differentiate into myoblasts, and proliferate. They either fuse with themselves, damaged muscle fibres, or remain quiescent as satellite cells at the sarcolemma. Satellite cells are characterised by expression of the muscle-specified paired box (Pax) transcription factor Pax7 [13]. They also consist of a majority of Myf5+ cells which act as an initiator of myogenic differentiation, marking the commitment of this cell population to the myogenic lineage [14].

Satellite cell usage has been promising. Studies have demonstrated their ability to regenerate large parts of musculature in vivo with low tumourgenic potential [15, 16]. Extracellular factors are necessary for the function of the satellite cell, and ex vivo studies have shown rapid de-differentiation after a few cell cycles [17]. These cells have potential for the treatment of muscular dystrophy. Early studies in mice lacking the gene for dystrophin production, showed that an injection of normal satellite cells into the muscle belly resulted in fusion with host fibres and extensive production of dystrophin [18]. Later studies however showed an immune response to the satellite cells and poor survival [19]. More efficient methods of delivery have been researched including transplanting individual muscle fibres (containing at least seven satellite cells) or isolating “purer” sources of satellite cells. Although some of these studies have shown promising results, the inability of these cells to cross the endothelial cell wall makes systemic delivery impossible, which impacts on their use to heal diseased diaphragm and cardiac muscles [20]. 

**Figure 1:**
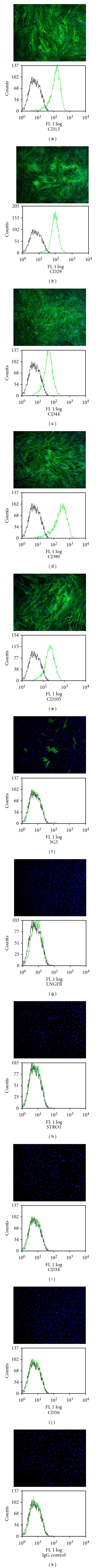
Cell surface epitope characterisation of passage 2 infrapatellar fat-pad-derived stem cells using a panel of antibodies. Cell surface staining using FITC-conjugated secondary antibody (green) and DAPI (blue) shows that the cells stain strongly for CD13, 29, 44, 90, and 105, and poorly for 3G5, LNGFR, STRO-1, and CD34 and 56. No staining was observed for the IgG control. The staining pattern is confirmed by flow cytometry and shows the increase in fluorescence (green) compared with the autofluorescence (black) [21].

MSCs can be obtained from a variety of different sources which can harbour myogenic potential. The first evidence of this was reported in 1998 in transgenic mice, showing that transplantable progenitors in bone marrow could be recruited to injured muscle and take part in repair [22]. Many studies have shown their potential in differentiating ex vivo into skeletal muscle under the right conditions [23–25]. Some studies have shown a low incorporation rate of MSCs into myofibres [26]. MSCs can however impose an additional paracrine effect on differentiation and tissue regeneration via cytokine pathways [27]. MSCs, unlike satellite cells maintain their stem-cell characteristics when systemically delivered and pass through vascular walls into target tissues [28]. There are a number of other tissue sources of stem cells for skeletal muscle engineering which are summarised in Table 1. MSCs are recognised using a range of cell surface markers as shown in Figure 1 [21]. 

## 4. Matrices

In vivo, the extracellular matrix of muscle provides fibres with the architecture to support development and function. It is a highly organised tissue with high cell density, with the parallel orientation of muscle fibres generating longitudinal contraction [29]. During tissue engineering, therefore, a scaffold is needed to mimic this matrix. In vivo studies have shown that stem cells with extracellular matrix, when injected into damaged muscle such as gastrocnemius, can significantly improve functional recovery when compared to matrix alone [30]. There are many different permutations to matrix structure and material. Matrix structure can be two-dimensional or three-dimensional. Scaffold material can be biodegradable or nonbiodegradable. Biodegradable matrices can be synthetic or natural. There are relative advantages and disadvantages to each.

The extracellular matrix of muscle in vivo is three-dimensional. Traditional cell culture has made use of 2D (two-dimensional) surfaces for ex vivo cell growth and is valuable in identifying cell structure and differentiation. In such environments, cells are forced to adopt unnatural characteristics, including aberrant flattened morphologies. 2D culture is not suitable for engineering 3D muscle tissue. Advantages of 3D over 2D culture include enhanced proliferation and differentiation of stem cells. In addition, 3D culture is more likely to accurately reflect the in vivo tissue environments from which cultured cells are derived.

Recent research on 2D “cell sheet technology” has shown promise, however, using temperature-responsive 2D scaffolds made out of a polymer, poly (N-isopropylacrylamide). Cell layers (with their extracellular matrix) can separate out with increasing temperature, obviating the need for enzymes [31]. Parallel alignment of fibres can be reached by techniques such as “electrospinning” and “microgrooving.” Microgrooving uses abrasives to create microgrooves within the matrix and has shown promising results in the orientated cell growth of myoblasts [32]. Electrospinning technique uses electrical current to form fibres as well as proteins of the natural extracellular matrix and can uniquely mimic the structure of the natural extracellular matrix [33]. Out of these 2D cell sheets, 3D matrices can be made, from 2D layering on a vascular bed. One disadvantage of cell-sheet technology is the inability of myoblasts to proliferate and differentiate more than 150 micrometres from a nutrient source [34]. Also, the electrospinning of nanofibres can often lead to them being densely packed, which can lead to poor cell infiltration [35]. 

The vast majority of scaffolds developed are biodegradable. When these degrade, remodelling to the natural muscular extracellular matrix can occur [36]. 3D scaffolds made from synthetic material such as polylactic-co-glycolic acid (PGA) fibre mesh sheets can provide rigidity and connection [37]. The nano- and microscale features of a polymer scaffold cause alignment of myoblasts and cytoskeletal proteins and promote myotube assembly to mimic the organisation seen in skeletal muscle. The surface stiffness in the polymer can help in the differentiation of satellite cells [38]. Parallel alignment can be induced by applying a strong magnetic field or mechanical strain [39]. 

Natural biodegradable 3D scaffolds also contain aligned topographical features causing alignment of myoblasts and proteins. Fibrin can be used, mixed with satellite cells and a growth medium. The original fibrin matrix is eventually taken over by the muscle progenitor cells, which produce their own extracellular proteins. Fibrin has the advantage of being able to bind growth factors such as IGF-1. In vitro models have been encouraging, showing that normal skeletal muscle in structure and function can be produced [40]. Collagen has also been used as a biodegradable 3D scaffold in some studies to good effect [41]. The type of proteins used for the scaffold is important. A recent study in mice showed that stem cell proliferation and differentiation using laminin and Matrigel was superior to collagen type 1, fibronectin, and gelatin [42]. 

In summary, although a pluripotent cell source is desirable, the tumour-forming potential in the use of these cells at present likely represents an unacceptable risk. Therefore, taking into account the literature discussed, a satellite cell source in a 3D matrix with a biodegradable scaffold appears to be the current optimum method of skeletal muscle tissue engineering.

## 5. Clinical Applications

Clinical trials on human subjects are limited due to the difficulties encountered with satellite cells, and the myogenic potential of alternative progenitor cells, delivery methods of these cells and the search for the “ideal” matrix. Highlighted below are the main clinical findings from human trials.

### 5.1. Muscular Dystrophies

These are a group of heterogeneous disorders producing progressive weakness, muscle wasting, and in the case of Duchenne muscular dystrophy usually paralysis and death in the patient's early 20s. Traditional treatment was limited to pharmacological suppression of the immune response with cortocosteroids.

Since the 1990s, from the first clinical trial in humans, it has been shown that stem cell transplantation via intramuscular injection can lead to dystrophin production. In 1991, Law injected extensor digitorum brevis (EDB) muscle in each of three Duchenne muscular dystrophy (DMD) boys with myoblasts. He demonstrated increases in isometric twitch and voluntary contractions whereas sham-injected EDBs showed reductions. All patients expressed dystrophin in their muscles following injection [43]. The next landmark study by Law involved 21 patients, with intramuscular injections of myoblasts. At 3 months, of the 69 muscle groups tested for isometric force generation in these subjects, 43% showed a mean increase of 41.3%. Eighty-one percent of the muscles tested showed either an increase in strength or did not show continuous loss of strength [44]. 

Several other studies began to question the longevity of muscle function following the intramuscular injections. Karpati et al. [45] showed that 12 months following multiple intramuscular bicep injections in 8 patients, only 3 had improved muscle strength. Tremblay et al. [46] showed in 5 patients that one month after myoblast transplantation into tibialis anterior (TA), the percentage of dystrophin-positive fibres ranged from 0–36%, compared to 0–4% on the control side. The expression of dystrophin in these fibres was generally low and most likely less than 10% of the normal level. In the biceps brachii on both sides 6 months after the transplantation, less than 1.5% of dystrophin-positive fibres were detected. No patients had improved strength at followup.

Mendell et al. injected donor myoblasts once a month for six months into the biceps brachii muscle of one arm in 12 boys with Duchenne's muscular dystrophy. Six months after treatment, there was no significant difference in muscle strength between the arms injected with myoblasts and sham-injected arms. In one patient, 10.3 percent of muscle fibers expressed donor-derived dystrophin after myoblast transfer. Three other patients also had a low level of donor dystrophin (<1 percent); eight had none [47]. 

Neumeyer et al. evaluated myoblast implantation therapy in three subjects with Becker muscular dystrophy. Each patient received 60 million myoblasts implantated into one TA muscle. They had begun cyclosporine immunosuppression two months prior to implantation and this was continued for 1 year. Results showed that myoblast implantation did not improve strength of the implanted TA muscles [48]. Skuk et al. showed similarly disappointing results after myoblast transplantation in the TA of 9 patients with the percentage of myofibers expressing donor's dystrophin varying from only 3.5% to 26% at 4-week follow-up [49].

Several other studies have shown a similar trend, but with no significant improvement in muscle function [50]. One of the problems with intramuscular injections for systemic conditions is the need to perform large numbers of injections to target different areas of muscle in order to gain a clinical response. Secondly, as already highlighted, vital muscles, such as the diaphragm for respiration, are not suitable for this form of treatment.

It was shown by Gussoni et al. [51] in mice that a marrow-derived cell could migrate into areas of muscle degeneration, undergo myogenic differentiation, thereby participating in muscle repair. Systemic delivery obviously holds the advantage of negating the need for multiple injections into the muscle belly, although an immune response to these cells is possible. Recent developments in the field of gene transfer therapy promise hope for future treatment possibilities. Cassano et al. recently showed that electrotransfer of “Magic”-Factor-1 gene into adult mice promoted muscular hypertrophy, improved running performance, and accelerated muscle regeneration after injury [52]. Phase I trials after gene transfer in patients with Duchenne muscular dystrophy have shown no adverse events [53]. 

It is likely that a strategy for treatment of these disorders will require a combination of stem cell and gene transfer techniques and we await the results in a few years time from ongoing trials.

### 5.2. Heart Failure

Like skeletal muscle cells, myocardial cells are striated; containing actin and myosin filaments arranged in the form of sarcomeres. They differ in that they interconnect through gap junctions to transfer electrical impulses. Muscle-derived myoblasts are considered an optimal cell therapy for heart failure, as they can be easily obtained from the same patient, rapidly expanded in vitro, and transplanted back into the patient's heart [20]. 

Several randomised controlled trials have shown benefits after transepicardiol injections of skeletal myoblasts [54–56]. Patients have benefited through an increased left ventricular ejection fraction, end-systolic volume, and subsequent symptomatic improvement. Concern remains about the increased occurrence of ventricular tachycardia following treatment. Ex vivo studies have shown embryonic stem cells to be of value in the development of new myocardial tissue [57].

### 5.3. Stress Urinary Incontinence

Stress urinary incontinence (SUI) is characterised by the loss of small amounts of urine when intra-abdominal pressure increases through laughing, coughing, or exercising. Muscle, connective tissue, and nerve damage during childbirth appears to be the most important risk factor [58]. Traditional treatment options of pelvic floor muscle training, pharmacological agents, and surgical solutions have had limited success. Recently, stem cell treatment has focused on treating the connective tissue and skeletal muscle component of the rhabdosphincter—thought to be the structure most important in controlling continence [59]. 

Most clinical trials in humans have involved muscle-derived stem cells injected under transurethral ultrasound guidance, together with a fibroblast/collagen suspension followed by pelvic floor exercises and transvaginal electrical stimulation [60, 61]. Numerous studies have shown benefit in females with stress incontinence, showing one-year cure rates up to 93% [60–63]. These patients have shown increased electromyelogram activity in the rhabdosphincter and increased urethral thickness. There have been reported benefits of using autologous-derived adipose stem cells in some patients [64]. Studies have also shown benefits of using stem cells in men for postprostatectomy stress incontinence [65]. 

## 6. Summary

Stem cells are emerging as a potential source of tissue repair and regeneration in many musculoskeletal tissues [66–80]. Although most advances have been made with bone, cartilage, tendon, and ligaments [81–109], this review shows that the application of stem cells in skeletal muscle regeneration following injury and disease is slowly emerging. Although satellite cells have attracted much interest due to their commitment to the myogenic lineage, their ability to cross the endothelial junction is limited, thus meaning locallydelivered transplantations are required. An appropriate matrix is needed to cultivate stem cells prior to their delivery in vivo. Human trials thus far have concentrated mainly on patients with muscular dystrophies, heart failure, and stress urinary incontinence. While successful results have been shown in patients treated with myoblast transplantation in heart failure and urinary incontinence, stem cell use in muscular dystrophies has so far been limited. Recent studies using gene therapy in combination with stem cell transplantation has shown some promise. Treatment using stem cells for skeletal muscle regeneration should combine a systemically delivered progenitor cell with controlled differentiation into myoblasts in vivo which can cross the endothelial lining of the blood vessel and target damaged muscle. We look forward to future studies developing current techniques and highlighting potential uses in the regeneration of skeletal muscle following trauma and disease.

## Figures and Tables

**Table 1:** Potential of other cell sources for skeletal muscle engineering.

Cell type	Source	Potential advantages
Skeletal muscle side population	Skeletal muscle	Can be delivered systemically May have increased capacity to incorporate into stem cell of muscle

Muscle-derived stem cells	Skeletal muscle	Can undergo myogenic and osteogenic differentiation Can repopulate haematopoietic lineage

Mesoangioblasts	Other mesodermal tissues, for example, dorsal aorta	Can be delivered systemically May be able to efficiently regenerate normal skeletal muscle

Pericytes	Basement membrane adjacent to endothelial cells Pancreas, adipose tissue, placenta	May improve the physiological performance of skeletal muscle Can be easily manipulated in vitro to reduce host's immune response

Adipocytes	Adipose tissue	Proven good differentiation into myogenic cells in vitro and in vivo

Embryonic stem cells and induced-pluripotent stem cells		Can regenerate acutely and chronically injured muscle but concerns of tumourigenic potential and ethical concerns
